# Sperm DNA Fragmentation and Sperm-Borne miRNAs: Molecular Biomarkers of Embryo Development?

**DOI:** 10.3390/ijms24021007

**Published:** 2023-01-05

**Authors:** Anna Chiara Conflitti, Gaia Cicolani, Alessandra Buonacquisto, Francesco Pallotti, Fabiana Faja, Serena Bianchini, Giovanna Blaconà, Sabina Maria Bruno, Antonella Linari, Marco Lucarelli, Diletta Montanino, Ludovico Muzii, Andrea Lenzi, Francesco Lombardo, Donatella Paoli

**Affiliations:** 1 Laboratory of Seminology–Sperm Bank “Loredana Gandini”, Department of Experimental Medicine, “Sapienza” University of Rome, 00161 Rome, Italy; 2 Department of Experimental Medicine, “Sapienza” University of Rome, 00161 Rome, Italy; 3 Department of Maternal Infantile and Urological Sciences, “Sapienza” University of Rome, 00161 Rome, Italy; 4 Pasteur Institute, Cenci Bolognetti Foundation, 00161 Rome, Italy

**Keywords:** miR-34c-5p, miR-449b-5p, Sperm DNA Fragmentation, sperm-borne miRNA, Intracytoplasmic Sperm Injection, embryo development

## Abstract

The evaluation of morpho-functional sperm characteristics alone is not enough to explain infertility or to predict the outcome of Assisted Reproductive Technologies (ART): more sensitive diagnostic tools are needed in clinical practice. The aim of the present study was to analyze Sperm DNA Fragmentation (SDF) and sperm-borne miR-34c-5p and miR-449b-5p levels in men of couples undergoing ART, in order to investigate any correlations with fertilization rate, embryo quality and development. Male partners (n = 106) were recruited. Semen analysis, SDF evaluation and molecular profiling analysis of miR-34c-5p and miR-449b-5p (in 38 subjects) were performed. *Sperm DNA Fragmentation evaluation*- a positive correlation between SDF post sperm selection and the percentage of low-quality embryos and a negative correlation with viable embryo were found. SDF > 2.9% increased the risk of obtaining a non-viable embryo by almost 4-fold. *Sperm miRNAs profile*—we found an association with both miRNAs and sperm concentration, while miR-449b-5p is positively associated with SDF. Moreover, the two miRNAs are positively correlated. Higher levels of miR-34c-5p compared to miR-449b-5p increases by 14-fold the probability of obtaining viable embryos. This study shows that SDF, sperm miR-34c-5p, and miR-449b-5p have a promising role as biomarkers of semen quality and ART outcome.

## 1. Introduction

What is a “healthy” spermatozoon? It is a “viable” male gamete able to reach the fertilization site and contribute to early embryonic development [1] thanks to its specific structural, morpho-functional and molecular properties. Spermatozoa carry DNA and other molecules, such as centrioles, mitochondria, proteins, and sperm RNAs, that are important in the different stages of reproduction, from spermatogenesis to embryonic development. The latest edition of the World Health Organization (WHO) manual highlights the importance of sperm chromatin integrity not only for fertilization potential but above all for embryonal implantation and development and for pregnancy rates in natural and assisted reproduction [2]. Sperm chromatin has a unique, complex structural organization that reduces the size and shape of the nucleus and makes it inaccessible to external agents. This chromatin organization is necessary for successful sperm function and any change can cause delays or defects in the delivery of paternal DNA. Sperm DNA Fragmentation (SDF), in the form of single- or double-strand breaks, is one of the most common types of damage affecting the nucleus. From a clinical perspective, sperm DNA damage has been associated with impaired spermatogenesis and infertility, and can have negative consequences on the reproductive process [3]. 

For years, sperm was considered as nothing more than a vehicle for the transport of genetic material to the oocyte. It is now known that it provides both a genetic and an epigenetic contribution to fertilization as a consequence of an increased awareness the biological relevance not only of sperm DNA, but also of sperm RNA. During spermiogenesis, various mRNAs are produced and then sequestered into “ribonucleoprotein particles” before transcriptional quiescence. Most of these RNAs are lost as cytoplasmic droplets, but a small, complex population is preserved in a non-random manner in the mature spermatozoon. The transcripts that remain inside the sperm thus comprise of a selected source of coding and nc (non-coding) RNAs, including both fragmented and degraded mRNAs: si (small interfering), mi (micro), pi (Piwi-interacting) and lnc (long non-coding)-RNAs [4]. Evidence that non-coding RNAs appear during the final phases of spermatogenesis or are acquired during epididymal passage suggests that they may be involved in post-fertilization events [5]. Maturation of the male gamete in the epididymis can be considered as the quality control phase ensuring a healthy spermatozoon [1]. The most abundant miRNA in the male gamete, miR-34c, has been described as essential for the first cell division in mouse zygotes [6] and has been correlated with the success of Intracytoplasmic Sperm Injection (ICSI) [7]. In this case, miR-34c is often associated with miR-449b, part of the miR-449a/b/c family. Although these miRNAs are from different families with different transcriptional loci, they have similar sequences and hence share both targets and functions. Members of both families are co-expressed in the testes and they are both downregulated in various sperm disorders [8]. Here, miR-449b, which is highly expressed in sperm, improves the first cleavage division, epigenetic reprogramming and apoptotic status of embryos [9]. Its expression has also been correlated with sperm motility, morphology and concentration [10]. The transcripts preserved in the sperm could therefore provide a way to assess the congruity of the molecular events occurring during spermatogenesis, and could also have an active role after fertilization. This latter point is highly important, as the use in Assisted Reproductive Techniques (ART) of sperm with epigenetic marks from infertile men could increase the risk of transmitting undesirable epigenetic modifications to the next generation [5]. 

The above evidence shows that SDF and sperm miRNAs are important molecular aspects involved in the reproduction [11]. New and more specific biomarkers would be useful in clinical practice to improve the work up of the infertile couple for counseling purposes. In light of this, SDF, sperm miR-34c and miR-449b would appear to have a promising role as biomarkers of seminal quality and ART outcome. The aim of the present study was to analyze SDF and sperm miR-34c-5p and miR-449b-5p levels in men from couples undergoing ART, in order to investigate any correlations with fertilization rate, embryo quality and embryonic development. This study could contribute to knowledge of the molecular pathways of spermatogenesis, fertilization, and embryogenesis and the identification of possible clinical biomarkers that could help improve ART.

## 2. Results

### 2.1. Demographic Characteristics

Among infertile couples referring to the Department of Maternal Infantile and Urological Sciences, “Sapienza” University of Rome, Policlinico Umberto I Hospital, to undergo ICSI cycles, 106 male partners were recruited. The mean age of the female partner was 37.0 ± 3.8 years and that of the male partner 41.0 ± 6.0 years. 

The main reasons for referring to ART Center were the presence of a female factor (either ovulatory or tubal factor) in 60/106 couples (56.6%), a male factor for 21/106 couples (19.8%), factors in both partners (couple infertility) in 7/106 (6.6%), couple’s infertility and idiopathic infertility in 18/106 (17.0%). Table 1 summarizes demographic characteristics of the couples and Anti-Müllerian Hormone (AMH) value on the day of oocyte pick-up. 

For ART, semen samples of male partners were processed by Swim-up (SW) or Density Gradient Centrifugation (DGC) and then were used in 106 ICSI cycles: a total of 477 oocytes were recovered by stimulation, 396 of which were inseminated, 292 oocytes were fertilized (fertilization rate 73.7%). Of the fertilized oocytes, 136 did not develop into a viable embryo (46.6%) and 156 embryos (52 top quality, 76 good quality, 28 low quality) were transferred in 85 women (mean 1.9 ± 0.7 embryos transferred per woman) between days 3 and 6.

### 2.2. Sperm DNA Fragmentation (SDF) Evaluation

#### 2.2.1. Semen Parameters

**Figure 1:**
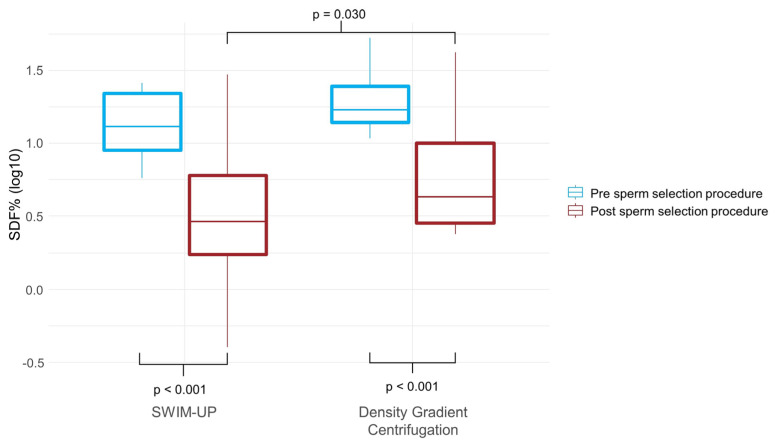
Comparison of SDF% before and after sperm selection procedures by DGC and SW.

The mean semen characteristics of the male partner were above the fifth percentile of the WHO 2010, while only 20.8% of patients (22/106) were below this value (Table 2). Semen samples after SW or DGC showed significantly better motility and morphology than at baseline (Table 2). Table 2 indicates, also, a significant reduction in SDF of samples post sperm selection procedures, which appears to be greater post SW compared to selection on DGC (SDF post DGC vs post SW: 11.1 ± 10.8% vs 4.5 ± 6.1%; *p* = 0.030, Mann Whitney U test) (Figure 1). Table 3 shows a negative correlation between SDF (pre and post sperm selection) and sperm concentration, progressive motility and normal morphology (pre and post sperm selection) (Table 3a,b).

#### 2.2.2. ICSI Outcome

Considering the ICSI outcome, significant differences were observed between the progressive motility of the seminal samples post sperm selection, the ages of both partners and the BMI of the male partner compared to the success of oocyte fertilization. No differences were found regarding SDF (Table 4).

**Figure 2:**
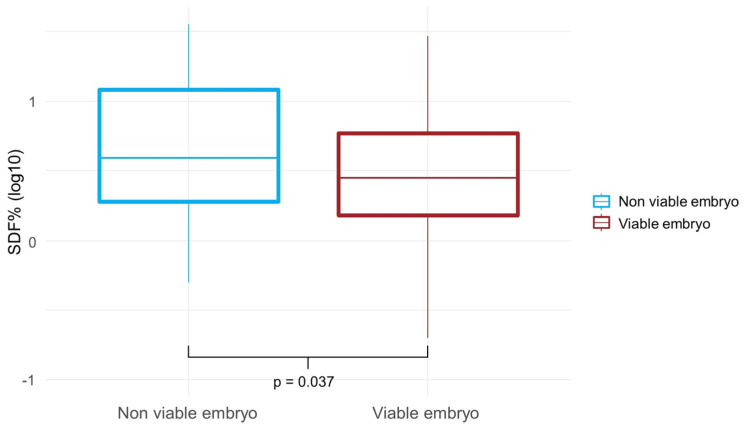
SDF post sperm selection procedures in semen samples according to the formation of a viable embryo (*p* = 0.037).

SDF post sperm selection procedures were significantly increased in the case of non-viable embryo (8.3% ± 8.0 vs. 4.8 ± 5.5 %, SDF in non-viable embryos vs. SDF in viable embryos, respectively; *p* = 0.037) (Figure 2). By calculating Spearman correlation coefficients, a positive trend was observed between post-sperm selection SDF and the percentage of low-quality embryos developed after insemination and a negative correlation with the embryo development (Table 5).

Linear and logistic regression models were constructed in order to validate the previously described associations between SDF post sperm selection and viable embryos. The univariate model showed a significant association between SDF post sperm selection procedures and n° of viable embryo, independently of the age of the partners, selection method used and AMH levels of the female partner (Table 6). 

Finally, the weight of sperm DNA fragmentation on the development of embryo, was assessed by logistic regression (Table 7). The median SDF pre sperm selection was 14.4% and the median SDF post sperm selection was 2.9% and, for the purposes of regression analysis, the population was divided above and below these cut-offs. It was observed that only SDF post sperm selection was found to be significantly associated with the development of embryo. In particular, SDF > 2.9% increased the risk of non-viable embryo by almost 4-fold (OR 3.95, 95%CI 1.088–14.379, *p* = 0.037). 

### 2.3. Sperm miR-34c-5p and miR-449b-5p

In a subgroup of 38 patients a molecular evaluation of sperm miR-34c-5p and miR-449b-5p was performed. Table 8 show demographic characteristics of the couples of the subpopulation studied. In particular, the mean age of the male partner was 39.8 ± 5.2 years with a mean BMI of 25.5 ± 3.5 kg/m^2^.

#### 2.3.1. Semen Parameters

**Figure 3:**
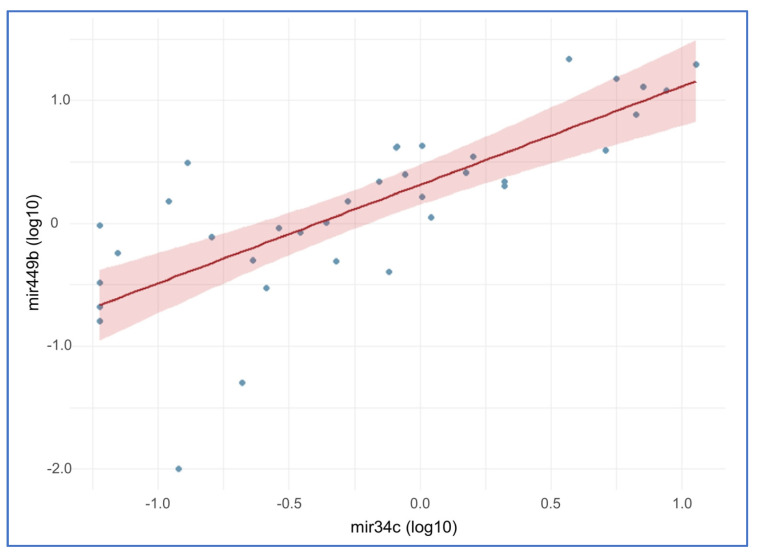
Correlation between the two miRNAs.

The seminal characteristics, SDF and miRNAs levels of male gametes post sperm selection of this study subpopulation are summarized in Table 9. Table 10 shows the correlations between the investigated miRNAs and seminal quality. Both miRNAs correlated positively with the sperm concentration of the sample. miR-449b-5p correlated significantly and positively with sperm DNA fragmentation (Table 10). Moreover, a strong correlation was found between the two miRNAs (Table 10, Figure 3). Univariate statistical models confirmed a positive association between miR-449b and SDF (*p* = 0.044), while no association was found between the two miRNAs and age, BMI or smoking (Table S1).

#### 2.3.2. ICSI Outcome

**Figure 4:**
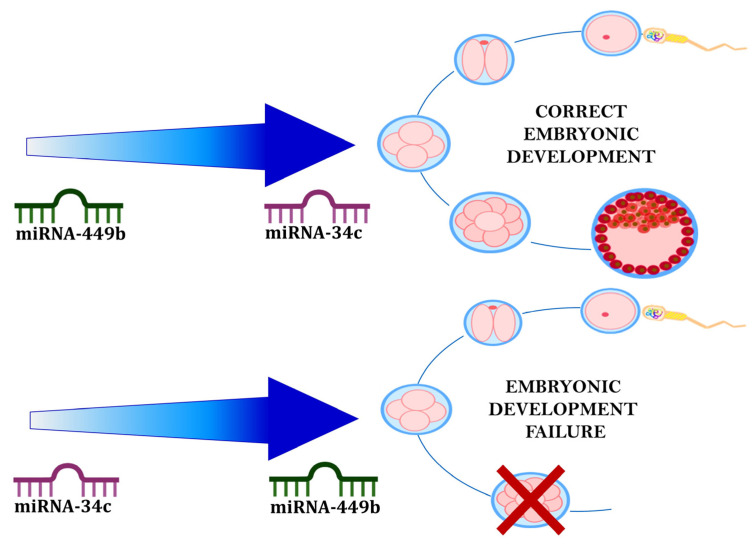
Hypothesis of the combined effect of the investigated miRNAs (miR-34c-5p and miR-449b-5p) on the embryonic development. The color intensity in the arrows corresponds to different behavior of miR-449b-5p and miR-34c-5p in the spermatozoon. The upper arrow represents the condition where there is a lower level of miR-449b-5p compared to miR-34c-5p; the lower arrow corresponds to the condition where there is a lower level of miR-34c compared with miR-449b. The lighter part of the arrow corresponds to lower levels of a miRNA compared to the other and the darker part indicates the increased level.

Logistic regression models were constructed in order to identify whether the sperm-borne miRNAs considered are potential predictors of positive ICSI outcome (viable embryo), “adjusting” by female variables (AMH). In particular, there is an association of the two miRNAs regarding embryonic development. It was observed that an increase in the expression of miR-34c-5p adjusted for miR-449b-5p is associated with an approximately 14-fold increase in the probability of obtaining viable embryos (OR 14.266, 95% CI 1.328-153.221, *p* = 0.028). On the other hand, higher levels of miR-449b-5p reduced the probability of the event (OR 0.038, 95% CI 0.002-0.847, *p* = 0.039) (Table 11) (Figure 4).

## 3. Discussion

### 3.1. Sperm DNA Fragmentation (SDF)

#### 3.1.1. Sperm DNA Fragmentation and Seminal Parameters

In reproductive biotechnology, considerable importance has been turned to the sperm DNA integrity, which is critical for proper transmission of genetic material to the embryo. High sperm DNA fragmentation has been associated with alterations in spermatogenesis with possible negative consequences on male reproductive potential [12]. Several works have shown that SDF evaluated by Terminal deoxynucleotidyl transferase dUTP nick end labeling (TUNEL) assay can be a potential fertility biomarker to distinguish of infertile patients from men with proven fertility [13,14]. Hichri et al. (2018) have shown DNA fragmentation has been positively correlated with percentage of abnormal forms and negatively with the sperm concentration and sperm motility [15]. Our data confirm the correlation between SDF and seminal parameters both before and after sperm selection procedures. There is a strong negative correlation of SDF with seminal quality in terms of sperm concentration, progressive motility, and normal morphology. In addition, a significant improvement in the percentage of spermatozoa with DNA damage was found after the sperm selection with a significant reduction in SDF after SW compared with DGC. This confirms literature data, as the increase of SDF is higher after DGC respect to Swim up suggesting a preferential use of the latter procedure to select spermatozoa for ART. The mechanism that induces the increase in SDF during DGC selection remains unclear. It has been hypothesized that heavy metal contamination present in the colloidal silicon gradients could induce a localized oxidative stress that promotes DNA breaks in samples selected by DGC [16].

#### 3.1.2. Sperm DNA Fragmentation and ICSI Outcome

DNA damage can affect embryo development, implantation, and pregnancies in both natural and assisted reproduction, and has been particularly associated with recurrent miscarriages. Several papers have also shown a correlation between sperm DNA fragmentation and poor embryo quality [17,18,19,20]. Borges et al. found that SDF was significantly correlated with slower rate of cleavage speed, poor embryo quality at day 3, poor blastocyst development, and implantation but did not identify any correlation with fertilization and pregnancy [19]. Our data also indicate that the fertilization rate depends on the couple’s age and BMI but not on DNA damage. Indeed, a significant and positive association between SDF post sperm selection and non- viable embryos was detected, independently of the age of the partners, the sperm selection procedures used, and the AMH levels of the female partner. Similar to the literature data, a positive correlation between SDF post sperm selection and the percentage of low-quality embryos and a negative correlation with viable embryo formation was found in the present study. In particular, an SDF>2.9% increased the risk of obtaining a non-viable embryo by almost 4-fold. Our data confirm that sperm DNA fragmentation evaluation could be informative during the infertile couple’s work up in order to improve counseling for couples undergoing ART.

### 3.2. Sperm-Borne miRNA

#### 3.2.1. Sperm miR-34c-5p and miR-449b-5p and Spermatogenesis

For a long time, it was believed that the spermatozoon was a simple carrier of the paternal genome because of the marked chromatin condensation and reduced amount of cytoplasm. Recent studies have shown that the male gamete contains several intrinsic factors that could be essential for fertilization and embryo development, among them are sperm proteins, sperm organelles, sperm mRNA, and sperm ncRNA [21]. A cluster of mRNAs has been identified that is common to both the sperm and the testis, supporting the hypothesis that numerous RNAs are produced during spermatogenesis and may be indicators of the functionality of this process [22,23]. It was also discovered that spermatozoa contain a set of transcripts that are not found in the oocyte but are present in zygotes. This finding validates the hypothesis that the male gamete delivers a set of RNAs to the oocyte during fertilization [24]. Through RNA-seq it has been found that the RNA pool in the male gamete consists of coding (mRNA) and ncRNA [25,26]. Four different species of RNAs can be distinguished in spermatozoa: mRNAs that are residues from the process of spermatogenesis with no known function in the oocyte [27]; mRNAs that originate from spermatogenesis with a potential function in the oocyte [28]; mRNAs classified as “foreign” RNAs as a result of their acquisition by spermatozoa during passage through the epididymis and from contact with fluids from accessory sex glands [29]; and ncRNAs acquired during the later stages of spermatogenesis or during post-testicular maturation that may have a potential function at the time of fertilization [30]. The 1% of sperm RNAs correspond to sncRNAs, among which around 7% of total sperm sncRNAs is composed of miRNAs. The miRNAs are small single-stranded ncRNAs consisting of 20-25 nucleotides that regulate gene expression by repressing the translation or cleaving the target mRNA [31]. The miRNAs present in the spermatozoon, called sperm-borne miRNAs, appear to be involved in controlling several processes of reproductive biology such as spermatogenesis, oocyte fertilization and embryo development [32,33]. In this regard, dysregulation of these miRNAs could lead to alterations in seminal fluid and be a cause of infertility. Several studies have demonstrated differential expression of sperm miRNA profiles between fertile and impaired spermatogenesis patients. In humans, the miR-34c is the most abundant sperm-borne miRNA [34] and is highly conserved among different species such as humans, mice and pigs [35]. This miRNA belongs to the miR-34 family, which includes three homologs miR-34a, miR-34b, and miR-34c [21]. While miR-34a is ubiquitously expressed, miR-34b and miR-34c are testis-specific [7]. It has been shown that miR-34c is involved in cell cycle control through modulation of cyclin, cyclin dependent kinase (CDK), p53 and Bcl-2 expression. In the male reproductive system this miRNA inhibits proliferation, promotes germ cell survival, differentiation, and apoptosis of excess and defective germ cells [36]. The implication of miR-34c in spermatogenesis is demonstrated by low levels of this sperm-borne miRNA associated with reduced sperm count, reduced motility and increased abnormal forms [21]. In animal models [37] and in humans [38] was reported correlation between seminal miR-34c-5p and sperm concentration, total motility, and normal morphology. The expression of miR-34c-5p is significantly lower in spermatozoa of men with several cases of spermatogenic impairment: oligozoospermia, asthenozoospermia, teratozoospermia, oligoasthenoteratozoospermia, idiopathic male infertility and also in seminal plasma of obstructive and nonobstructive azoospermia [8,39,40,41]. The miR-34c profile is often associated with miR-449b, which belongs to the miR-449a/b/c family. In the miR-449 family, miR-449b seems to have greater prominence because it is highly expressed in the spermatozoon [9]. The miR-34c and miR-449b belong to two different clusters and are transcribed from different loci but have similarities in sequence and therefore share targets and functions. Both are co-expressed in the testis and are downregulated under different conditions of spermatogenic impairment [8]. Several studies have suggested that alterations in the expression of miR-34c and miR-449b family members negatively affect male reproductive potential because of their involvement in biological processes such as spermatogenesis, fertilization, and embryo development [21,36,42]. No association of these two miRNAs with age, BMI and smoking was found in the present study, but it was observed that the expression levels of miR-34c-5p and miR-449b-5p in spermatozoa correlated positively with sperm concentration. These data agree with several studies suggesting that in humans altered expression of miR-34/449 family members are strongly associated with altered seminal parameters [36]. The influence of miR-34c-5p and miR-449b-5p on seminal quality confirms their role in spermatogenesis and suggests their role in multiciliated cell differentiation. In the male reproductive system, altered miR-34/449 family expression leads to defective ciliogenesis in efferent ductules epithelium, which can result in testicular dysfunction and agglutination of spermatozoa with subsequent impairment of sperm concentration. At the same time, alterations in ciliogenesis impair flagellum formation resulting in increased abnormal forms and reduced sperm motility [36]. Of considerable interest is the positive association between sperm miR-449b-5p and SDF. This suggests not only the involvement of this miRNA in altering spermatogenesis, but confirming the relevance of miR-449b-5p target genes associated with apoptosis, cell proliferation, and differentiation.

#### 3.2.2. Sperm miR-34c-5p and miR-449b-5p and ICSI Outcome

The discovery of RNAs transmitted from the spermatozoon to the zygote has opened new frontiers in the study of sperm-borne miRNA. Increasing evidence has indicated the potential contribution of miR-34c and miR-449b in the molecular regulation of embryo development, fertilization and pregnancy outcome by both natural and Assisted Fertilization. In the mouse model, the absence of miRNAs acquired by sperm in the caudal tract of the epididymis causes inhibition of implantation. This demonstrates that the final sperm-borne miRNA repertoire is not defined during spermatogenesis, but is modified during the passage of sperm into the male genital tract [43]. Liu et al. showed that levels of miR-34c comparable to those found in the spermatozoon are found in the zygote, but are not detectable in the embryo. This allows for the hypothesis that this miRNA is useful in fertilization and early embryonic development, but not in later stages [6]. To date, only three studies have investigated the relationship between sperm miR-34c levels, embryo development and ICSI outcomes. Shi et al. showed that miR-34c in spermatozoa is negatively associated with embryo development kinetics and positively associated with the percentage of blastocyst formation, high-quality blastocysts, and pregnancy [35]. Similarly, Cui et al. (2015) showed that sperm-borne miR-34c was associated with better embryo quality at day 3 and higher implantation, pregnancy, and birth rates [7]. Recently, Yeh et al. (2022) did not observe association between miR-34b/c and fertilization rate. However, they found that miR-34b/c levels in spermatozoa above a certain threshold were associated with increased implantation and pregnancy rates and decreased miscarriage rates, indicating better quality of early embryos [44]. In agreement with these studies, our data confirm that increased miR-34c-5p in spermatozoa is associated with a higher probability of obtaining viable embryos. This finding could indicate a possible role for miR-34c-5p as a biomarker predictor of ICSI outcome. In humans more difficulties are present in the evaluation of miR-449b-5p because its role *in vivo* or in ART has not yet been studied. Abu-Halima et al. (2014) found downregulation of miR-449b in testicular biopsies of infertile subjects [39]. In vitro experiments have shown that sperm miR-449b-5p plays important roles in the kinetics of cleavage, blastocyst formation, epigenetic reprogramming, and blastomere apoptosis [9]. Our study appears to be the first in the literature to analyze the expression of miR-449b-5p in spermatozoa related to the outcome of ART. Although the expression of sperm miR-34c-5p e miR-449b-5p are strongly and positively correlated in the whole caseload, our data reveal that the behavior of miR-449b-5p may be slightly different from that of miR-34c-5p. A relative increase of miR-34c-5p in spermatozoa compared with miR-449b-5p is associated with a 14-fold increase of the probability of obtaining viable embryos. In contrast, an increase in sperm-borne miR-449b-5p relative to miR-34c-5p reduces this probability and increases the risk of embryonic development failure. The latter result reinforces the evidence of the association of miR-449b-5p with SDF and apoptosis, cell proliferation, and differentiation. In agreement with other studies [45,46], our data show a direct association between the levels of miR-34c-5p and miR-449b-5p in spermatozoa, probably because they have similar sequences, targets, and functions [47,48]. Interestingly, in the model mouse, the ablation of miR-449a/b/c causes overexpression of miR-34b/c to compensate for the miRNA-449 deficiency [46,49]. The existence of this association allows to speculate on a possible synergistic action between the two miRNAs, the molecular mechanisms of which is unknown, but which would be interesting to study in the future. Rah et al. (2017) found that the miR-449b-5p rs10061133 polymorphism was associated with an increased risk of pregnancy loss in Korean women. Therefore, the presence of a polymorphism could modulate the expression of miR-449b-5p influencing its role in embryo development and pregnancy [50]. Furthermore, to explain the different behavior of these two miRNAs it should be emphasized that miR-449b and miR-34c might be subject to different regulation. Studies on neoplastic cells have shown that transcription factor E2F1 can cause the expression of miR-449 members in a p53-independent way, whereas p53 can upregulate the expression of miR-34 family members [51,52,53] by controlling cell differentiation and triggering the apoptotic process through two different pathways that are independent of each other and regulated by different genes [53]. This could also explain our data about the association of sperm miR 449b-5p with sperm DNA damage. Consequently, the expression and function of sperm miR-34c and miR-449b could affect embryo development differently depending on the prevailing signaling pathway. Another molecular mechanism that could explain our data could be the differential methylation of CpG in promoter regions of genes coding for miR-34c or miR-449b. Two recent studies reported that men with idiopathic male infertility and altered seminal parameters showed hypermethylation of promoter region of miR-34b-5p [10] and miR-449b [41] with consequently a significant reduction in expression levels of these miRNAs in sperms. Interestingly, patients with oligoasthenoterazoospermia (OAT) had the highest percentage of methylation as well as the lowest miR-34c-5p and miR-449b levels [10,41]. The reasons for the inappropriate methylation of CpG in their promoter regions are still poorly understood. Smoking has been described as a factor leading to abnormal methylation of the miR-449 cluster promoter [10] but can be add many other parameters such as lifestyle, physical activity, stress and aging that could affect the DNA methylome in spermatozoa.

## 4. Materials and Methods

### 4.1. Patients

The study was approved by the Ethics Committee “Sapienza” (Prot. 0810/2022). Written informed consent was obtained from all study participants. 

For this study were recruited the male partner of 106 infertile couples referred to the Department of Maternal Infantile and Urological Sciences, Sapienza University, Policlinico Umberto I Hospital, to undergo ICSI cycle. The patients had not been medically or surgically treated in the 3 months before the study and did not have any conditions (fever, etc.) that might interfere with the semen analysis. 

The inclusion criteria for the couples selected were:

- age over 18 years and under 50 for male partners;
- age over 18 years and under 43 for female partners;
- presence of mature oocytes suitable for insemination;
- possibility of taking a sufficient aliquot of semen pre and post sperm selection procedures for sperm DNA fragmentation and miRNAs analyses.

The exclusion criteria were:

- presence of endometriosis diagnosis for female partners;
- presence of recurrent pregnancy loss;
- presence of andrological pathologies (cryptorchidism, clinically relevant varicocele, hypogonadism, etc.) and/or endocrinological diseases which may interfere with semen quality;
- azoospermia (both obstructive and non-obstructive);
- genetic syndromes and/or abnormal karyotype;
- previous treatment(s) with chemo/radiotherapy and/or potentially gonadotoxic drugs for oncological pathology;
- previous treatment(s) with potentially gonadotoxic drugs for non-oncological pathologies.

Semen analysis, Sperm DNA Fragmentation (SDF) evaluation, and analysis of miR-34c-5p and miR-449b-5p levels in spermatozoa were performed in Laboratory of Seminology–Sperm Bank “Loredana Gandini”, Department of Experimental Medicine, “Sapienza” University of Rome. Assisted reproductive procedures were performed in the ART Center by a single embryologist.

### 4.2. Semen Analysis

Semen samples were collected by masturbation after 2–7 days abstinence. All samples were allowed to liquefy at 37 °C for 60 min and were then assessed according to WHO (2010) [54]. The following variables were taken into consideration: volume (mL), total sperm number (n × 10^6^ per ejaculate), progressive motility (%), and morphology (% abnormal forms).

### 4.3. Sperm Selection Procedures

Depending on the quality of basal semen parameters the most appropriate sperm selection procedure was chosen for each sample. Sperm selection was performed according to the clinical practice at the ART Center.

- Swim-up (SW): the semen sample was diluted 1:1 with a culture medium (Multipurpose Handling Medium—MHM; Fujifilm, USA) pre-warmed to 37 °C and centrifuged at 1200 rpm for 10 min. At the end of centrifugation, the supernatant was removed and then 100–300 µL of culture medium pre-warmed to 37 °C and containing 10% human albumin was layered on the pellet. After a one-hour incubation at 37 °C in 5% CO_2_, the upper layer was removed to retrieve the spermatozoa most suitable for insemination.
- Density Gradient Centrifugation (DGC): equal volumes of 80% gradient, 40% gradient (MHM; Fujifilm, USA) and finally the seminal fluid was stratified in a conical test tube. The tube was centrifuged for 10 min at 1500 rpm in order to obtain the sedimentation of sperm with the best microscopic characteristics due to the filtering action of the gradients. The pellet obtained at the end of centrifugation was suspended in culture medium and incubated for one hour at 37 °C in 5% CO_2_ and then used to inseminate the oocytes.

### 4.4. Assisted Reproductive Procedures

ICSI cycle management consisted of down regulation of endogenous hormones with a short protocol with a GnRH antagonist (Fyremadel 0.25 mg, Ferring) starting from day 2 of treatment cycle with administration of 150 IU urinary FSH + LH (Meropur, Ferring). From the sixth day of stimulation, daily monitoring of follicles size by US was performed and plasma levels of E2 and progesterone were measured. The criteria used for triggering ovulation with 10.000 IU hCG (Gonasi HP 5000, ^®^Ibsa) s.c. were plasma E2 between 1000 and 3000 pg/mL and at least four follicles >17 mm mean diameter (two perpendicular measurements) with plasma progesterone <1.5 ng/mL.

Oocyte retrieval was performed 36 h after hCG administration, by transvaginal US-guided follicular aspiration under intravenous sedation. Selection of samples for our study was performed according to the Italian law regulating ART at the time of the pick-up (law 40/2004 and subsequent modifications).

At the time of follicular aspiration, MII oocytes surrounded by expanded cumulus-corona (CC) cells were identified by phase contrast microscopy (PCM) and rinsed with a buffer. Only MII oocytes that appeared of good quality when observed by PCM were selected for light/electron/confocal microscopy observations. They should have the following: a rounded, regular shape; a clear, moderately granular cytoplasm; a narrow perivitelline space (PVS) with the 1st polar body (PBI); and an intact, colorless zona pellucida (ZP) [55]. The resultant embryos were scored according to established criteria [56].

Briefly, Day-3 embryos were scored on the basis of morphologic appearance of cells, fragmentation and multinucleation:

- Grade 1 embryos: stage-specific cell size, <10% fragmentation and no multinucleation;
- Grade 2 embryos: stage-specific cell size for majority of cells, 10–25% fragmentation and no multinucleation;
- Grade 3 embryos had cell size not stage specific, >35% fragmentation and evidence of multinucleation.

The scoring system for blastocysts is a combination of the stage of development and of the grade of the inner cell mass (ICM) and of the trophectoderm (TE) degree of expansion of the blastocyst (grade from 1 to 4); good, fair and poor ICM and TE (grade 1 or A, 2 or B, 3 or C) in consideration of size. 

Embryo in which development has been arrested for at least 24 h, or in which all the cells have degenerated or lysed was defined as a non-viable embryo [56].

Ultrasound guided embryo transfer took place 72–120 h following insemination.

The luteal phase was supported with the daily vaginal administration of 600 mg of progesterone.

### 4.5. Sperm DNA Fragmentation (SDF)

SDF was evaluated using TUNEL assay (Roche, In Situ Cell Death Detection Kit, Fluorescein, Roche, Basel, Switzerland). An aliquot of samples pre and post sperm selection was centrifuged and processed as previously described by Paoli et al. 2019 [57]. The samples were then analyzed under fluorescent microscope (Leica DMR; Leica, Wetzlar, Germany), counting at least 500 cells.

### 4.6. MicroRNA Analysis

Osmotic shock: an aliquot of semen samples post sperm selection was diluted with PBS and underwent osmotic shock to ensure the elimination of possible non-gamete cells. The method used was as described in Paoli et al. 2017 [58]. Samples were centrifuged at 13,000 rpm for 15 min. The pellets were resuspended with 1 ml of cell lysis buffer (0.1% SDS, 0.5% Triton X-100 and distilled H_2_O) and incubated for 60 min at +4 °C. After incubation, the absence of round cells was confirmed by optical microscopy. Next, the samples were centrifuged at 13,000 rpm for 15 min. Finally, the supernatants were removed and the pellets were used for RNA extraction.

RNA extraction and cDNA synthesis: total RNA was extracted from spermatozoa using the miRNeasy Mini Kit (Qiagen, Hilden, Germany). The RNA concentration was calculated by spectrophotometry using the NanoDrop ND-2000 (Thermo Fisher Scientific, Waltham, MA, USA). Reverse transcription for cDNA synthesis was carried out on 10 ng of RNA extracted from each sample in a final reaction volume of 15 μL according to the manufacturer’s instructions (microRNA RT kit -Applied Biosystem).

Digital PCR (ddPCR): the expression of miR-34c-5p and miR-449b-5p in spermatozoa was evaluated by single ddPCR. The reaction mix was composed of 1.5 μL of cDNA, 11 μL of 2 × ddPCR Supermix for probes (no dUTP) (Bio-Rad, Hercules, California, USA), and 1 μL of 20× TaqMan assay specific for each miRNA analyzed (Thermo Fisher Scientific, Waltham, MA, USA, Applied Biosystems -Assay ID hsa-miR-34c-5p: 000428; Assay ID hsa-miR-449b-5p: 001608). The reaction mix was loaded into the droplet generator cartridge. Next, 70 μL of droplet generation oil for probes was added at specific wells in the cartridge. The cartridge was transferred to the QX200 droplet generator. After droplet generation, the droplets (40 μL) were loaded at 96-well ddPCR plate and the plate was heat sealed with specific aluminum foil with the PX1 PCR plate sealer (Bio-Rad, Hercules, California, USA). Thermal cycling conditions were as follow: 95 °C for 10 min, then 40 cycles of 94 °C for 30 s and 60 °C for 1 min and one final step 98 °C for 10 min for the deactivation of the enzyme. Finally, the plate remained to 10 °C for 4 h to enhance dye stabilization.

### 4.7. Statistical Analysis

The statistical analysis was carried out using the software R version 3.4.2 (R Foundation for Statistical Computing, Vienna, Austria). Continuous variables are expressed either as mean ± standard deviations or median and interquartile range, based on the shape of distributions evaluated with the Kolmogorov–Smirnov test. Comparisons of variables before and after sperm selection technique were computed through paired sample t test or Wilcoxon signed-rank test, as appropriate. Categorical variables, expressed as frequencies or percentages, were confronted using the Fisher’s exact test. The correlations were computed using Spearman’s rank correlation test. A two-tailed p value lower than 0.05 was considered as statistically significant. Univariate linear models and logistic regression models have been constructed. Each relevant parameter (number of viable embryos for linear model. Viable and non-viable embryo for logistic regression models) was included as a dependent variable. Other variables (namely age, BMI, smoking status, sperm selection procedure, %SDF, miRNAs levels and partner’s AMH) were included in the model as independent variables. 

## 5. Conclusions

**Figure 5:**
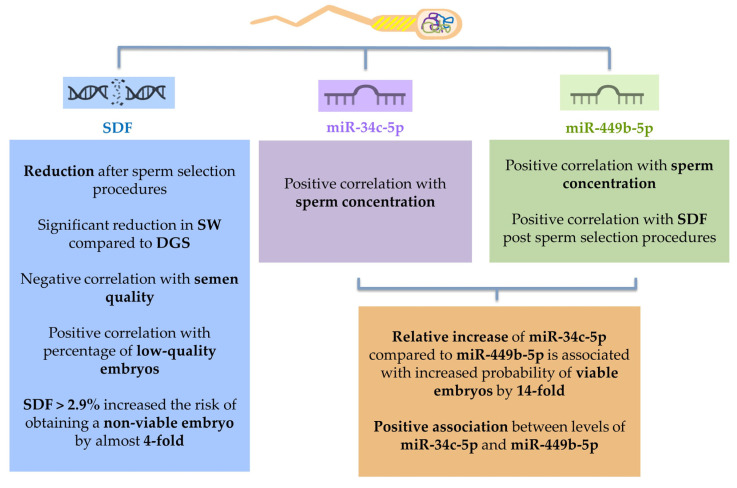
Summary of the study results.

This study arises from the need to identify accurate and sensitive diagnostic tools that can improve the counseling of infertile couples. Therefore, the aim was to identify possible new biomarkers in terms of sperm chromatin integrity and levels of sperm miR-34c-5p and miR-449b-5p in order to identify a possible impact on the outcome of ART. SDF, sperm miR-34c-5p and miR-449b-5p have been detected to be essential for spermatogenesis, successful fertilization and early embryonic development (Figure 5). The developing spermatozoon is affected by environmental changes and acquires certain molecular profiles that may compromise reproductive health. Some factors, such as diet, physical activity, mental stress, and exposure to certain substances, can be "stored" in the spermatozoon as epigenetic information that can change pre-implantation metabolism, oxidative stress, and subsequent embryo quality in vitro. Therefore, the health and quality of sperm delivered at the time of fertilization play an important role [59]. It is important to know the molecular aspect and function of sperm-specific intrinsic factors. These data demonstrate the promising role of SDF, sperm miR-34c-5p and miR-449b-5p as biomarkers of semen quality and ICSI outcome to improve the diagnostic/therapeutic workup of infertile couples.

## Figures and Tables

**Table 1:** The demographic characteristics of the couples and AMH value on the day of oocyte pick-up. Continuous variables are presented as mean ± SD and median (in italics); categorical variables are presented as counts and percentages.

	Age	BMI	Smokers	AMH [ng/mL]
♀	37.0 ± 3.8 *37.0*	22.5 ± 3.6 *22.1*	26/106 (24.5%)	2.0 ± 1.6 *1.6*
♂	41.0 ± 6.0 *40.0*	25.7 ± 3.1 *25.8*	33/106 (31.1%)	//

**Table 2:** Comparison of semen parameters before and after sperm selection procedures. Continuous variables are presented as mean ± SD and median (in italics); categorical variables are presented as counts and percentages.

	Sperm Concentration (×10^6^/mL)	Progressive Motility (%)	Abnormal Form (%)	SDF (%)	Below 5th Percentile WHO 2010
BASELINE 106 pz	50.9 ± 40.9 *40.0*	39.9 ± 16.5 *45.0*	90.8 ± 3.8 *90.0*	15.5 ± 8.8 *14.4*	22/106 (20.8%)
POST DGC 30 pz	18.9 ± 17.1 *13.0*	68.2 ± 32.9 *90.0*	51.0 ± 21.0 *46.5*	11.1 ± 10.8 ^a^ *6.2*	//
POST SW 76 pz	29.9 ± 19.5 *30.0*	89.4 ± 10.2 *90.0*	42.4 ± 10.7 *40.0*	4.5 ± 6.1 ^a^ *2.5*	//

^a^*p* < 0.001 vs. baseline—Wilcoxon signed rank test. DGC—Density Gradient Centrifugation; SW—Swim up.

**Table 3:** Spearman correlations between SDF with seminal characteristic: (**a**) pre sperm selection procedures; (**b**) post sperm selection procedures.

**(a)**				
		**Pre Sperm Selection Procedures**		
		**Conc./mL**	**Progressive Motility (%)**	**Abnormal Form (%)**
SDF PRE sperm selection procedures	Spearman’s ρ *p* value	−0.413 <0.001	−0.508 <0.001	0.416 <0.001
**(b)**				
		**Post Sperm Selection Procedures**		
		**Conc./mL**	**Progressive Motility (%)**	**Abnormal Form (%)**
SDF PRE sperm selection procedures	Spearman’s ρ *p* value	−0.364 <0.001	−0.223 0.036	0.365 <0.001

**Table 4:** The seminal characteristics post sperm selection procedures and demographic characteristics are presented as mean ± SD and median (in italics), according to oocyte fertilization success (Mann Whitney U-test).

	Sperm Concentration (×10^6^/mL)	Progressive Motility (%)	Abnormal Forms (%)	SDF (%)	Age ♀	Age ♂	BMI ♀	BMI ♂
Fertilization	26.5 ± 19.5 20.0	85.1 ± 18.8 90.0	43.8 ± 13.2 40.0	5.2 ± 5.8 2.9	36.8 ± 3.8 37.0	40.5 ± 6.3 40.0	22.4 ± 3.6 22.1	25.5 ± 3.1 22.5
NO Fertilization	29.3 ± 19.5 36.0	57.1 ± 39.9 80.0	60.0 ± 26.8 52.0	10.1 ± 11.0 5.6	40.3 ± 2.4 41.0	48.1 ± 6.7 47.0	23.1 ± 3.8 23.5	28.7 ± 3.0 29.3
*p* value	*0.760*	*0.002*	*0.073*	*0.380*	*0.015*	*0.005*	*0.541*	*0.015*

**Table 5:** Spearman correlations between SDF pre and post sperm selection procedures with ICSI outcome.

		Number of Inseminated Oocytes	Number of Fertilised Oocytes	Low-Quality Embryos Ratio *	Viable Embryo
SDF% pre sperm selection procedures	Spearman’ρ *p* value	−0.043 0.706	−0.101 0.378	0.094 0.472	−0.132 0.245
SDF% post sperm selection procedures	Spearman’ρ *p* value	−0.081 0.483	0.020 0.865	0.230 0.074	−0.238 0.036

* Percentage of low-quality embryos/total embryos.

**Table 6:** Coefficients, significance, partial frame age and observed power of the univariate linear model analysis (dependent variable: viable embryos).

	Coeff.	95% CI	*p* Value
SDF pre sperm selection procedures (%)	0.881	−0.337–2.099	0.153
SDF post sperm selection procedures (%)	−0.742	−1.292–−0.193	0.009
Age ♀	−0.026	−0.093–0.040	0.430
Age ♂	0.026	−0.016–0.068	0.223
Selection technique (DCG vs. SW)	0.217	−0.303–0.737	0.407
AMH [ng/mL]	0.163	0.026–0.301	0.021

**Table 7:** Odds Ratio (OR), confidence interval (95% CI) and standard error (SE) from logistic regression models. (dependent variable: non-viable embryos).

	OR	95% CI	SE	*p* Value
SDF > 14.4% Pre-sperm selection	1.099	0.313–3.852	0.640	0.883
SDF > 2.9% Post-sperm selection	3.955	1.088–14.379	0.659	0.037
AMH	1.143	0.777–1.683	0.197	0.497
Age ♀	1.086	0.884–1.333	0.105	0.433
Age ♂	1.022	0.909–1.149	0.060	0.714
BMI ♀	0.933	0.772–1.129	0.097	0.477
BMI ♂	1.012	0.827–1.238	0.103	0.908
Selection technique (DCG vs. SW)	0.438	0.087–2.198	0.823	0.316

**Table 8:** Demographic characteristics of the couples of the subpopulation studied and AMH value on the day of oocyte pick-up. Continuous variables are presented as mean ± SD and median (in italics); categorical variables are presented as counts and percentages.

	Age	BMI	Smokers [%]	AMH [ng/mL]
♀	36.3 ± 4.6 *37.0*	22.5 ± 3.4 *22.2*	9/38 (23.7%)	2.2 ± 1.7 *1.9*
♂	39.8 ± 5.2 *40.0*	25.5 ± 3.5 *25.3*	14/38 (36.8%)	/

**Table 9:** Summary characteristics of molecular study subpopulation post selection. Continuous variables are presented as mean ± SD and median (in italics).

	Sperm Concentration (×10^6^/mL)	Progressive Motility (%)	Abnormal Form (%)	SDF (%)	miR-34c-5p [Copies/µL]	miR-449b-5p [Copies/µL]
POST sperm selection	33.1 ± 17.9 *37.0*	90.4 ± 1.8 *90.0*	41.7 ± 10.3 *40.0*	5.0 ± 6.8 2.9	1.8 ± 2.7 *0.7*	3.8 ± 5.5 *1.6*

**Table 10:** Spearman’s correlation between miR-34c-5p, miR-449b-5p, and seminal characteristic.

	Sperm Concentration(×10^6^/mL)	Progressive Motility (%)	Abnormal Form (%)	SDF (%)	miR-34c-5p [copies/µL]	miR-449b-5p [copies/µL]
miR-34c-5p	0.468 0.003	0.099 0.556	0.218 0.188	0.274 0.117	//	0.812 <0.001
miR-449b-5p	0.514 <0.001	0.078 0.652	0.235 0.167	0.362 0.035	0.812 <0.001	//

**Table 11:** Odds Ratio (OR), confidence interval (95% CI) and standard error (SE) of logistic regression to evaluate the association between viable embryo and the expression of miR-34c-5p and miR-449b-5p (dependent variable: non-viable embryos).

	OR	95% CI	SE	*p* Value
miR-34c-5p	14.266	1.328–153.221	1.211	0.028
miR-449b-5p	0.038	0.002–0.847	1.580	0.039

## Data Availability

The data underlying this article will be shared on reasonable request to the corresponding author.

## Supplementary Materials

The following supporting information can be downloaded at: https://www.mdpi.com/article/10.3390/ijms24021007/s1.
