# Supplementary material for: Sperm DNA Fragmentation and Sperm-Borne miRNAs: Molecular Biomarkers of Embryo Development?
Source: Int J Mol Sci. 2023 Jan 5;24(2):1007. doi: 10.3390/ijms24021007 (PMC9864861; doi:10.3390/ijms24021007)
Supplement: Supplementary file 1 [file ijms-24-01007-s001.zip › ijms-2100415-supplementary.pdf]

Supplementary Table S1. Univariate statistical models to evaluate the association between the parameters age, BMI, smoking and SDF on the expression of miR-34c-5p and miR-449b-5p.

		Univariate analysis		
		**B (95% CI)**	**B**	***p value***
Age ♂	miR-34c-5p	-0.035 (-0.078 – 0.008)	-0.266	0.107
	miR-449b-5p	-0.010 (-0.054 – 0.034)	-0.081	0.642
BMI ♂	miR-34c-5p	-0.027 (-0.092 – 0.038)	-0.137	0.410
	miR-449b-5p	-0.045 (-0.104 – 0.014)	-0.259	0.133
Smoking ♂	miR-34c-5p	-0.198 (-0.666 – 0.269)	-0.142	0.395
	miR-449b-5p	-0.309 (-0.741 – 0.124)	-0.245	0.156
SDF	miR-34c-5p	0.383 (-0.199 – 0.965)	0.230	0.190
	miR-449b-5p	0.520 (0.015 – 1.026)	0.364	**0.044**
